# Examining the influence of gender, education, social class and birth cohort on MMSE tracking over time: a population-based prospective cohort study

**DOI:** 10.1186/1471-2318-12-45

**Published:** 2012-08-13

**Authors:** Fiona Matthews, Riccardo Marioni, Carol Brayne

**Affiliations:** 1 MRC Biostatistics Unit, Cambridge, UK; 2 Department of Public Health and Primary Care, University of Cambridge, Cambridge, UK

**Keywords:** MRC CFAS, Population norms, Cohort effects, MMSE, Longitudinal, Cognitive decline

## Abstract

**Background:**

Whilst many studies have analysed predictors of longitudinal cognitive decline, few have described their impact on population distributions of cognition by age cohort. The aim of this paper was to examine whether gender, education, social class and birth cohort affect how mean population cognition changes with age.

**Methods:**

The Medical Research Council Cognitive Function and Ageing Study (MRC CFAS) is a multi-centre population based longitudinal study of 13,004 individuals in England and Wales. Using ten years of follow-up data, mean Mini-mental State Examination (MMSE) scores were modelled by age and birth cohort adjusting for non-random drop-out. The model included terms to estimate cohort effects. Results are presented for five year age bands between 65–95 years.

**Results:**

At a population level, women show greater change in MMSE scores with age than men. Populations with lower education level and manual work also show similar effects. More recent birth cohorts have slightly higher scores.

**Conclusion:**

Longitudinal data can allow examination of population patterns by gender, educational level, social class and cohort. Each of these major socio-demographic factors shows some effect on whole population change in MMSE with age.

## Background

Quality of life in old age is affected by many factors including maintenance or loss of cognitive ability. For successful cognitive ageing, particular attention has been paid to the potential protective effects of intellectual activity and good health status [1]. Whilst studies have analysed these factors as predictors of longitudinal cognitive decline, few have described their impact on the population distribution of cognition across age cohorts, and between groups with different characteristics.

Systematic reviews and individual studies have linked cognitive reserve factors such as education, occupation (a proxy measure of social class), and social engagement to a decreased risk of dementia and cognitive decline in later life [2-5]. In addition to these associations there is also biological evidence to link increased education to a decreased risk of dementia through a neurocompensation mechanism [6]. Among individuals with the same burden of post-mortem neuropathology, those with higher education have been shown to be less likely to have dementia in life [6].

However, while low education has been associated with poorer cognition it late-life, and in some cases, decline over two waves, when the same data have been analysed over multiple waves null findings have been observed [7]. There may also be measurement and cohort differences for the effects of education on cognition. Against this is recent evidence from the 10/66 Study team, which showed increased education to reduce the risk of dementia across six middle-income countries [2].

There is conflicting evidence whether cognitive change by age differs by sex. Some studies have found no evidence for a difference [8,9], others have reported 50% more decline amongst women [10,11]. There are inconsistent findings for a higher incidence and prevalence of dementia in women compared to men [12,13].

In the absence of cohort effects, population norms based on cross-sectional data could be used to describe population average scores. However, taking norms from cross-sectional data fails to utilise all of the information available from population-based longitudinal studies. This analysis employs methods that overcome these issues and calculates norms based on the complete data resource. Research has also suggested that more recent birth cohorts perform better [14]. Relatively little work has been carried out over prolonged periods in population representative samples. Studies with a long follow-up that have provided findings have tended to be volunteer cohorts [15] or specialised samples [16,17]. A previous study by our group presented Mini-Mental State Examination (MMSE) norms in a population-based sample of individuals aged 65 years and above [18]. This paper extends that work by providing a detailed discussion of the cohort, sex, education, social class, and centre (region of residence) effects on mean MMSE scores by age.

## Methods

Data stem from the Medical Research Council Cognitive Function and Ageing Study (MRC CFAS, http://www.cfas.ac.uk). CFAS, which began in 1989, is a population-based study of individuals aged 65 years and over from six centres in England and Wales [19]. Five of the centres had a standardised study design and are analysed here: Cambridgeshire (n = 2,601), Gwynedd (n = 2,625), Newcastle (n = 2,524), Nottingham (n = 2,514), and Oxford (n = 2,740). Cognition was assessed using the MMSE [20] at baseline and after two, six, and ten years of follow-up. The study sample is described in detail in Additional file 1: Appendix 1. MRC CFAS has multi-centre research ethics committee’s approval and ethical approval from the relevant local research ethics committees. Written informed consent for participation in the study was obtained from participants.

### Social class and education

Occupations were coded according to the Registrar General’s occupation-based social class divisions using Computer Assisted Standard Occupational Classification software [21]. Non-manual (I-IIINM) occupation was compared to manual (IIIM-V). Self-reported education was split into statutory or less (≤9 years) and more than statutory (10+ years).

### Statistical analysis

A detailed description of the analysis methodology has been reported previously [22,23]. Briefly, the methods account for all of the longitudinal data and adjustments are made for individuals who have missing information using inverse probability weighting. Individuals are initially regressed against missing the next longitudinal data point based on their age, sex, centre, living alone, education, study route to interview and MMSE seen at the last non-missing interview. The model is then expanded to estimate a regression coefficient for their missing MMSE score which has the effect of removing the need for the prior regression coefficient for MMSE from the model (when the coefficient in the logistic regression equals zero). The predicted probabilities from this model are then used to define the inverse probability weights. These weights are then combined with a generalisation weight back to the original population to create a complete weight per individual per interview.

These weights are then used within a standard cubic spline regression model with knots at ages 70, 75, 80, and 85. This was to allow a flexible but smooth relationship between mean MMSE and age (entered as a linear term). Terms for cohort effects were also estimated in the models; cohorts were defined by age group at baseline (65–69, 70–74, 75–79, 80–84, or 85+). Socio-demographic groups were not constrained to have the same cohort effect.

The mean was chosen as the main summary measure of the MMSE distribution. The analysis was replicated using the median and quantile regression. Results were similar although they did not always have unique solutions. All analyses were performed in STATA version 8 [24].

### Precision of estimates

A bootstrap approach was used to construct 95% confidence intervals (CI) around cohort effects and mean MMSE estimates [25]. One-thousand bootstrap samples were drawn randomly, conditional on the CFAS design i.e., the marginal totals of people at baseline above and below age 75 within each centre were constrained to be the same as the baseline sample. Weights were re-calculated and analyses were run on the resulting datasets.

## Results

**Figure 1:**
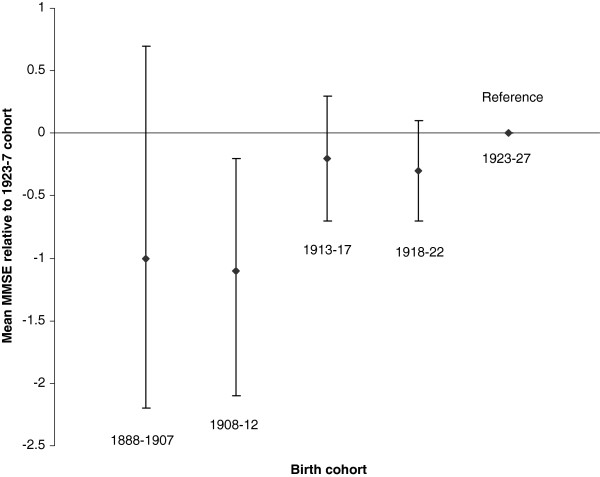
Cohort effects on mean MMSE.

The birth cohorts presented in Figure 1 correspond to people aged 65–69, 70–74, 75–79, 80–84 and 85+ years at baseline. Cohorts born before 1912 are estimated to score one point lower on average than the 1923–27 birth cohort, although the 95% CI ranges are wide. More recent birth cohorts received slightly more education; 38% of those from the 1923–27 cohort had more than 9 years of education compared to 33% of those born before 1908.

**Figure 2:**
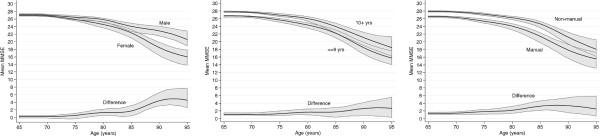
Mean MMSE (and 95% CI) for the birth cohort 1923–27 by sex, education, and social class.

There was a small difference at age 65 between the sexes with men scoring slightly higher than women (Figure 2 illustrating the 1923–7 birth cohort). Differences are illustrated at the bottom of each panel; significant differences are observed where the confidence interval about the mean difference does not include 0. The gap between mean MMSE for men and women increased by 4 points (95% CI: 1.3 - 6.8) by age 95.

The differences for the 1923–27 birth cohort for education and social class are also shown in Figure 2; Additional file 1: Appendix 2 illustrates the mean MMSE values and confidence intervals shown in Figure 2 for the latest birth cohort. There was a mean MMSE difference of about 2 points at all ages, favouring the more educated/non-manual workers. There was some evidence that the less educated/manual workers average score dropped at a faster rate, though this did not reach conventional levels of statistical significance. The average drop in MMSE score was 1.4 points more for those with ≤ 9 years of education compared to those with 10 or more years. For manual workers the average drop in MMSE was 1.1 points more than for non-manual workers.

The centre-specific estimates of mean MMSE and 95% CI are shown in Additional file 1: Appendix 3. Average scores dropped the most in Nottingham and the least in Oxford. The 95% confidence intervals about these estimates were relatively large and a Wald test on 4 degrees of freedom showed differences were not statistically significant after an adjustment for multiple testing. These findings mirror the previously reported incident and prevalent dementia results, where there was variability but no significant difference between centres [19].

## Discussion and conclusions

Changes over time in mean MMSE scores by birth cohort were estimated for the socio-demographic factors sex, education, social class and study centre. Differences were seen by sex with women’s average scores declining significantly faster with age than men’s. Null associations were observed for cognitive change by education, social class, and study centre. However, point estimates indicated that change was more marked for the less educated and for manual workers.

Challenging aspects for this study include adjustment for dropouts and accounting for the complex study design. This analysis does both simultaneously. Whilst cohort effects can be estimated from this study design, true cohort studies where new population samples are drawn at different time points, e.g., Seattle Longitudinal Study [14], can investigate these effects more rigorously as learning effects and dropouts do not potentially bias results. In this paper, cohort effects were extrapolated across the age range and calculated to represent those at the youngest possible study entrance age of 65 years. This means that due to there only being 10 years of follow-up, the trajectories of the youngest cohort at the oldest ages were based on the results from the older birth cohorts who were observed at those ages during the study.

Limitations of this work include using the MMSE as a measure of cognition. While it can be used as a measure of global cognitive function, it exhibits floor and ceiling effects [26]. The primary clinical role of the MMSE is as a screening instrument for dementia. Moreover, there are also strong links between MMSE scores and education [27]. In this analysis the difference in longitudinal MMSE trajectories split by education level was not statistically significant although those with less than statutory education did have lower mean MMSE point estimates at all ages. Finally, due to methodological limitations it was not possible to determine the overlap between the education and social class findings.

Integrating these findings with the literature is complex as results may vary depending on the cognitive test that is applied. Another limitation may be the inclusion of education as a binary variable (less than statutory versus statutory or more). Whether years of education, highest qualification obtained, actual school grades, or a composite measure would be more effective is unclear and beyond the scope of the current analysis.

Mortality and cognitive decline are competing risks for the older population. Individuals with a below average MMSE die earlier [28,29], which means that average population cognitive decline is not as great as average individual decline. If mortality acts on two groups in a similar manner, an increasing gap between population average cognition with age corresponds to one group experiencing more decline at the individual level. This assumption does not necessarily explain the differences in MMSE scores seen between the sexes in this study. Women live longer than men both with and without disability [30]. Hence, the force of mortality between the sexes appears to be different and an increasing gap at the population level does not necessarily imply individual change.

These findings mirror those reported in the literature. In analyses of cognitive decline, women declined significantly faster than men [10,11], and whilst not statistically significant, less educated and manual workers declined faster than more educated and non-manual workers [10,11]. The direction of the cohort effects replicates previous findings [31,32]. The minimal increase in years of education for more recent birth cohorts seems unlikely to be big enough to explain these cohort effects, although changes in the content of that education may play a role.

Examining cohort effects in a follow-up study is complicated enormously by non-random dropouts. Moreover, the calculation of population norms is often restricted to cross-sectional (baseline) data without utilising information from all available longitudinal follow-up waves. Alternative study designs are needed to provide definitive conclusions about cohort effects.

## Competing interests

There were no conflicts of interest for any of the authors.

## Authors’ contributions

FEM contributed to data acquisition, assisted in obtaining funding, analysed and interpreted data, drafted the first version of the manuscript and revised and edited the manuscript. FEM is guarantor of the analysis. REM contributed to design and revised and edited the manuscript. CB conceived the research idea, contributed to design, data acquisition, funding and revised and edited the manuscript. All authors read and approved the final manuscript.

## Authors’ information

The Medical Research Council Cognitive Function and Ageing Study website can be found at the following address: http://www.cfas.ac.uk.

## Pre-publication history

The pre-publication history for this paper can be accessed here:

http://www.biomedcentral.com/1471-2318/12/45/prepub

## Supplementary Material

Additional file 1 **Appendix 1.** Numbers of subjects in the analysis. Appendix 2: Mean MMSE scores and 95% confidence intervals for the 1923-27 birth cohort split by age and covariates (sex, centre, education and social class). Appendix 3: Mean MMSE (and 95% CI) by centre for the birth cohort 1923-27. Click here for file
