# Supplementary material for: Examining the influence of gender, education, social class and birth cohort on MMSE tracking over time: a population-based prospective cohort study
Source: BMC Geriatr. 2012 Aug 13;12:45. doi: 10.1186/1471-2318-12-45 (PMC3542122; doi:10.1186/1471-2318-12-45)

Appendix 1: Numbers of subjects in the analysis

Wave	Years after baseline	Interviews	Alive but not approached	In analysis	Dropout since last wave	Died since last wave
(Sampling frame)				16,525		
1	0	13,004		12,573	3,952	-
2	2	8,827		8,468	2,841	1 264
3	5	2,452 †	3 941*	2,221†	1,038	1 268
4	10	3,145		2,990‡	1,218	1 972

* These are people who were interviewed at incidence screen at wave 2 and were not approached for interview until wave 4.

† Includes all individuals alive in the Cambridgeshire centre, i.e. 1085 interviews, and 1040 MMSE scores.

‡ Includes 18 temporary refusers at wave 3

Appendix 2: Mean MMSE scores and 95% confidence intervals for the 1923-27 birth cohort split by age and covariates (sex, centre, education and social class)

		Age (years)					
		65	70	75	80	85	90
Sex	Male	27.0 (26.7, 27.2)	26.8 (26.5, 27.1)	26.2 (25.6, 26.8)	25.7 (25.0, 26.2)	24.1 (22.7, 25.1)	22.9 (21.4, 24.3)
	Female	26.7 (26.3, 26.9)	26.5 (26.2, 26.7)	25.7 (25.2, 26.2)	24.4 (23.5, 25.0)	22.4 (20.9, 23.2)	18.5 (16.7, 20.0)
Centre	Cambridgeshire	26.5 (26.1, 26.9)	26.4 (25.8, 26.8)	25.6 (24.9, 26.5)	24.5 (23.5, 25.3)	22.9 (21.5, 24.1)	20.8 (19.1, 22.3)
	Gwynedd	27.0 (26.5, 27.3)	26.8 (26.4, 27.1)	25.9 (25.1, 26.5)	24.8 (24.0, 25.6)	23.4 (21.9, 24.2)	18.8 (16.5, 21.6)
	Newcastle	26.8 (26.4, 27.2)	26.7 (26.4, 27.1)	26.2 (25.4, 26.9)	25.3 (24.4, 26.0)	23.0 (21.2, 24.1)	19.7 (17.0, 22.1)
	Nottingham	26.6 (25.8, 27.1)	26.4 (25.7, 26.8)	25.3 (24.1, 26.3)	23.4 (21.3, 24.7)	20.8 (17.7, 22.8)	16.6 (13.0, 19.4)
	Oxford	27.0 (26.7, 27.4)	26.9 (26.6, 27.2)	26.5 (25.0, 27.0)	26.0 (25.2, 26.6)	24.1 (22.3, 25.2)	21.8 (19.0, 23.7)
Education	< 9 years	26.4 (26.0, 26.6)	26.3 (25.9, 26.4)	25.4 (24.9, 25.9)	24.1 (23.4, 24.7)	22.1 (21.0, 23.0)	18.4 (17.0, 20.1)
	≥10 years	27.7 (27.3, 27.8)	27.5 (27.1, 27.6)	26.7 (26.2, 27.2)	25.9 (25.1, 26.4)	23.8 (22.3, 24.8)	21.0 (19.0, 22.9)
Social class	Manual	26.3 (26.0, 26.5)	26.0 (25.8, 26.3)	25.1 (24.6, 25.7)	23.9 (23.0, 24.5)	21.3 (19.8, 22.3)	17.7 (15.7, 19.1)
	Non-manual	27.7 (27.6, 27.9)	27.6 (27.5, 27.8)	27.1 (26.7, 27.4)	26.1 (25.5, 26.6)	24.7 (23.7, 25.4)	21.3 (19.6, 22.6)

Appendix 3: Mean MMSE (and 95% CI) by centre for the birth cohort 1923-27

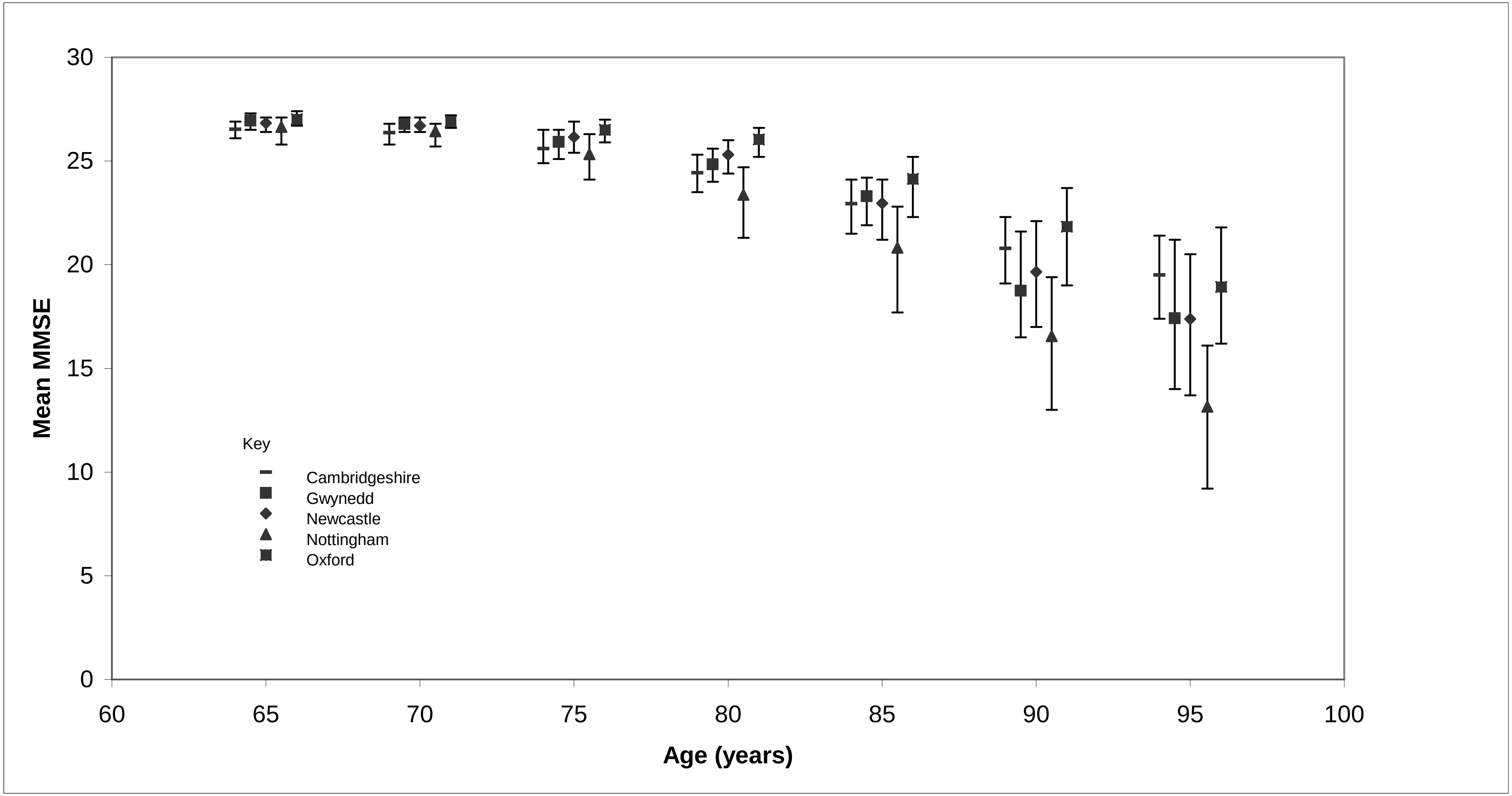

Supplement: Additional file 1 — Appendix 1. Numbers of subjects in the analysis. Appendix 2: Mean MMSE scores and 95% confidence intervals for the 1923-27 birth cohort split by age and covariates (sex, centre, education and social class). Appendix 3: Mean MMSE (and 95% CI) by centre for the birth cohort 1923-27. [file 1471-2318-12-45-S1.pdf]
